# Adherence to recommended diet among patients with diabetes mellitus type 2 on follow-up at Adama Hospital Medical College, Ethiopia

**DOI:** 10.3389/fmed.2024.1484071

**Published:** 2024-11-26

**Authors:** Saron Abose, Godana Arero Dassie, Abebe Megerso, Tesfaye Getachew Charkos

**Affiliations:** Department of Public Health, Adama Hospital Medical College, Adama, Ethiopia

**Keywords:** dietary, non-adherence, type 2 diabetic mellitus patients, Adama, Ethiopia

## Abstract

**Introduction:**

Non-adherence to dietary guidelines is a significant challenge in managing diabetes mellitus and its complications. Its consequences were significantly associated with a deterioration in patients’ quality of life and an increased socioeconomic burden on healthcare delivery systems. This study aimed to assess the magnitude of adherence to recommended diet and associated factors among patients with diabetes mellitus type 2 on follow-up care at Adama Hospital Medical College Oromia, Ethiopia.

**Methods:**

A hospital-based cross-sectional study design was conducted. Participants were selected through systematic random sampling. Data were collected using structured, interviewer-administered questionnaires. The perceived dietary adherence questionnaire was used to assess the level of dietary adherence. A simple binary logistic regression was used to identify candidate variables, while a multivariable logistic regression assessed factors associated with adherence to the recommended diet. A *p*-value <0.05 were considered as statistically significant. All analyses were performed using SPSS and R programming software.

**Result:**

A total of 405 participants were included in the study, with a response rate of 96.2%. The magnitude of non-adherence to the recommended diet was 64.2% (95% confidence interval [CI]: 59.8, 68.6). In the multivariable logistic regression model, patients with low and middle income (AOR = 8.0; 95% CI: 3.4, 19.2) and (AOR = 2.75; 95% CI: 1.49, 5.55) respectively, high glycemic level (AOR = 2.15; 95% CI: 1.17, 3.94), food insecure (AOR = 12.7; 95% CI: 5.79, 28.2), poor diabetic knowledge (AOR = 2.88; 95% CI: 1.49, 5.55) and low perceived susceptibility (AOR = 2.97; 95% CI: 1.62, 5.45) were significantly associated factors for non-adherence to recommended diet among patients with diabetes mellitus type 2.

**Conclusion:**

This study revealed that approximately two-thirds of patients with type 2 diabetes mellitus experienced non-adherence to the recommended diet. Key factors linked to dietary non-adherence among T2DM patients include low to middle income, elevated glycemic levels, household food insecurity, limited diabetes knowledge, and low perceived susceptibility. An integrated approach that combines socioeconomic support, nutritional guidance, and risk awareness may greatly enhance dietary adherence and optimize diabetes management.

## Introduction

Diabetes mellitus is a metabolic disorder with various causes that impair the body’s ability to process glucose for energy, leading to elevated blood sugar levels due to defects in insulin secretion, action, or both (1). It can significantly increase the risk of premature death (2). According to the World Health Organization, adherence is how a person’s behavior such as taking medication, following a diet, and making lifestyle changes aligns with their healthcare provider’s recommendations (3). Type 2 diabetes mellitus (T2DM) is a chronic metabolic disorder influenced by genetics, lifestyle, and diet (4) and represents 90% of all diabetes cases, posing a significant and growing global health burden (5). Managing Type 2 diabetes requires regular self-care and significant lifestyle changes, primarily dietary modifications (5). Diabetes patients who fail to adhere to the recommended dietary regimen are at risk of developing life-threatening complications (6).

Dietary non-adherence can progressively worsen diabetic conditions (7). Research indicates that the rates of dietary non-adherence among people with type 2 diabetes vary widely across countries, ranging from 29.9 to 97.5% (8, 9). Similarly, several studies suggested that dietary non-adherence rates among individuals with type 2 diabetes vary significantly, with 63% in Botswana, 97.5% in Egypt (3, 9), and 46.8 to 79% in Ethiopia (10, 11). The recommended diet includes fruits, vegetables, nuts, whole grains, and unsaturated fats, while snacks high in fat, sugar, or salt should be avoided (12).

A growing number of fast food restaurants, urbanization, globalization, and rapid socioeconomic development, which can lead to atypical consumption patterns and overdependence, may contribute to Type 2 diabetes mellitus patients’ difficulties in adhering to their dietary regimens (8). Non-adherence to diet can gradually worsen the disease, impacting the patient’s quality of life, the healthcare provider, and the healthcare delivery system. It can lead to increased mortality, longer hospital stays, and higher costs and burden of the disease (13).

Although a few studies in Ethiopia (14, 15) have used the 8-item Morisky Medication Adherence Scale (MMAS-8) to assess dietary adherence in patients with type 2 diabetes, this tool was originally designed to measure medication adherence rather than dietary habits. As a result, the MMAS-8 may not capture the full range of dietary practices essential for effective diabetes management. In contrast, our study utilizes the Perceived Dietary Adherence Questionnaire (PDAQ), a validated tool specifically developed to assess dietary adherence in alignment with the Canadian Diabetes Association (CDA) nutrition therapy guidelines (16, 17). The PDAQ’s validation against repeated 24-h dietary recalls provides a more accurate and focused measure of dietary adherence among patients with diabetes.

Additionally, our study includes a relatively larger sample size, enhancing the accuracy and precision of our findings. This robust methodological approach, combined with the larger sample, provides a more comprehensive understanding of dietary adherence patterns among Ethiopian patients with type 2 diabetes, offering valuable insights to guide more effective, population-specific dietary interventions.

## Methods

### Study setting and design

A hospital-based cross-sectional study was conducted at Adama Hospital Medical College from January 1 to February 14, 2023. This hospital, located in Adama town in the Oromia Region, is one of the first medical hospitals in the area, situated 100 km southeast of Addis Ababa. The hospital was inaugurated by missionaries from abroad in 1938 E.C. and was among the first non-governmental hospitals in the nation. It serves a catchment population of over 6 million people from five regions (Oromia, Amhara, Afar, Somali, and Dire-Dawa). The hospital was selected purposively for this study due to the availability of regular DM case follow-up and the large number of type 2 DM cases at the Diabetic Clinic.

### Inclusion and exclusion criteria for participants

This study included patients with Type 2 diabetes who had received treatment follow-up for at least 6 months during the data collection period. Patients with newly diagnosed Type 2 diabetes, those needing ongoing medical support and monitoring, and individuals under 18 years of age were excluded from the study.

### Sample size determination

A single population proportion formula was used to determine the sample size (18), assuming a 95% confidence interval (CI) with a 5% margin of error and a non-adherence proportion of 46.8%, based on a study conducted at Felege-Hiwot Referral Hospital in northwest Ethiopia (10). A total of 421 participants were included in the study, accounting for a 10% non-response rate.

n=Zα22p∗qd2=421

### Sampling procedures

A systematic random sampling method was used to select patients with type 2 diabetes mellitus (DM). The sampling interval was determined by dividing the total DM patient population by the calculated sample size. The first participant was chosen through simple random sampling, and subsequent participants were selected at regular intervals. Data were collected from each study participant during their clinic visits using a structured questionnaire administered through face-to-face interviews.

### Study variables and measurements

The dependent variable in this study was adherence to the recommended diet (categorized as adherent or non-adherent). The independent variables included various socio-economic and demographic factors, such as participant age, sex, educational status, residence, marital status, occupation, family income, and family size. Additionally, the study considered anthropometric and clinical characteristics, including BMI, glycemia levels, comorbidities, family history of diabetes mellitus (DM), duration of illness, treatment type, food-related factors, diabetes education, awareness of diabetic diets, and household food security status. Psychosocial and behavioral factors such as smoking and drinking habits, khat use, and family/social support were also included. Finally, the diabetic health belief model was assessed through perceived susceptibility, perceived severity, perceived benefits, perceived barriers, depression, and diabetic knowledge.

The study employed the Perceived Dietary Adherence Questionnaire (PDAQ) to assess dietary non-adherence. Developed by Asaad et al. (16), this nine-item tool measures patients’ perceptions of their adherence to dietary guidelines. Responses on the PDAQ are recorded on a seven-point Likert scale, reflecting food consumption over the past 7 days. Generally, higher scores indicate better adherence, except for items 4 and 9, which assess the consumption of high-sugar or high-fat foods; in these cases, higher scores signify lower adherence. A patient is considered to have good dietary adherence if they maintain a healthy diet at least 4 days a week.

The Perceived Dietary Adherence Questionnaire (PDAQ) (16) and the Morisky Medication Adherence Scale (MMAS) (19) both assess adherence but in different domains: PDAQ for dietary adherence and MMAS for medication adherence. The PDAQ has demonstrated strong validity and reliability, similar to the MMAS, with high internal consistency (Cronbach’s alpha >0.80) and stable test–retest reliability. Both tools are concise, reducing respondent burden and enhancing adequacy for clinical and research use. They are sensitive to adherence changes over time, making them valuable in intervention studies; however, the PDAQ is specifically tailored to assess dietary behavior changes.

Household food insecurity was assessed using the Household Food Insecurity Access Scale (HFIAS), adapted from the food and nutrition technical assistance guidelines. Responses were aggregated to create an index representing the level of household food insecurity (16, 20). Participants’ height was measured with a stadiometer, recorded to the nearest 0.1 cm, while their weight was measured using a digital scale, recorded to the nearest 0.1 kg. The scale was reset to zero before and after each measurement. Body mass index (BMI) was calculated as weight in kilograms divided by the square of height in meters (kg/m^2^).

The Patient Health Questionnaire-9 (PHQ-9) was utilized to assess depression in patients with Type 2 diabetes mellitus. This nine-item questionnaire was adapted from Pfizer Inc. (21). Diabetes knowledge was measured using a 20-item yes/no questionnaire, where each correct answer scored “1” and incorrect or unknown responses scored “0,” resulting in a total score ranging from 0 to 20. Participants with scores equal to or above the mean were classified as having good knowledge, while those scoring below the mean were considered to have poor knowledge (22). Diabetic health beliefs were evaluated using 14 adapted questions on a 5-point scale, based on a similar study conducted in Igala, Nigeria (23). These questions assessed participants’ perceptions of susceptibility and severity regarding diabetes complications, as well as the perceived benefits and barriers to dietary recommendations. The scores for each health belief construct were summed to categorize respondents as having high or low levels based on whether their scores were above or below the mean cutoff.

### Data quality assurance

Data collectors and supervisors received 1 day of training on the study tools, objectives, and interview techniques. Anthropometric measurements followed standard procedures with calibrated instruments, and regular supervision and follow-up were conducted. A pretest with 5% of type 2 diabetic patients from outside the study area ensured response consistency. The questionnaire was reviewed for content, consistency, and organization, and modified as needed. Data quality was monitored daily for completeness, consistency, and accuracy by the principal investigators.

### Data processing and analysis

Descriptive statistics, including frequencies and percentages for categorical variables and means with standard deviations for continuous variables, were used to summarize the data. Bivariate logistic regression analysis was conducted to examine associations between independent variables and adherence to recommended dietary practices, with variables showing a *p*-value less than 0.25 considered for multiple logistic regression to identify significant predictors of dietary adherence. Model fit for the final analysis was assessed using the Hosmer–Lemeshow test (*p*-value = 0.504), indicating an adequate fit. The Variance Inflation Factor (VIF) was calculated to evaluate multicollinearity, ensuring no interference with model interpretation, while the Shapiro–Wilk test assessed normality for continuous variables, confirming that assumptions for statistical analyses were met. A *p*-value of less than 0.05 was set to determine statistical significance. All analyses were conducted using SPSS version 26.0 and R version 3.4.3 (R Foundation for Statistical Computing, Vienna, Austria).

### Ethical clearance

Ethical clearance was obtained from the Adama Hospital Medical College of institutional review Board committee. Informed written consent was obtained from participants. Those unwilling to participate or who chose to withdraw during the study were allowed to discontinue their involvement at any time.

## Results

### Socio-demographic characteristics of the participants

Out of 421 eligible respondents, 405 (with a response rate of 96.2%) participated in the study. The mean age of participants was 56.2 years (SD ±10.11). Among the participants, 216 (53.3%) were female. Regarding religion, 242 (59.8%) participants were Orthodox, followed by Muslims 93 (23.3%). The majority of participants, 299 (73.8%), were married and 372 (91.9%) urban residents. One hundred thirty-three 133 (32.8%) participants had attended primary school, while 108 (26.7%) were illiterate. Of the total participants, 135 (33.3%) were housewives, 177 (43.7%) participants had medium monthly income, and 277 (68.4%) participants had family sizes of fewer than five members (Table 1).

**Table 1:** Socio-demographic and economic characteristics of patients with DM 2 type attending follow-up care at Adama Hospital Medical College, Oromia, Ethiopia, 2023 (*n* = 405).

Variable	Frequency	Percent (%)
Gender		
Female	216	53.3
Male	189	46.7
Age in year		
<39	18	4.4
40–49	98	24.2
50–59	128	31.6
>60	161	39.8
Religion		
Orthodox	242	59.8
Muslims	93	23.3
Others^a^	70	17.3
Residence		
Rural	33	8.1
Urban	372	91.9
Educational status		
No formal education	108	26.7
Primary	133	32.8
Secondary	102	25.2
College and above	62	15.3
Marital status		
Married	299	73.8
Widowed	49	12.1
Others^b^	57	14.1
Occupation		
Daily laborer	57	14.1
Employed	88	21.7
House wife	135	33.3
Retired	52	12.8
Others^c^	73	18.1
Monthly income in Birr		
Low	112	27.7
Medium	177	43.7
High	116	28.6
Family size		
<5	277	68.4
≥5	128	31.6

Other^a^: catholic, wakefeta; others^b^: single and divorced; others^c^: merchant, unemployment, and farmer.

### Anthropometric and clinical characteristics of study participants

The mean duration follow-up was 5.5 years (SD ±4.5), with 145 (35.8%) participants reported a family history of diabetes. Additionally, 188 (46.4%) participants had co-morbid conditions. A majority, 268 (66.2%), patients managed their blood glucose levels with oral hypoglycemic agents. Only 78 (19.3%) participants were members of the Ethiopian Diabetic Association. The mean of BMI was 24.2 (SD ±3.73), with 153 (37.8%) participants were classified as overweight and 23 (5.7%) as obese. Furthermore, 275 (67.9%) participants had fasting blood glucose levels greater than 126 mg/dL. Among the respondents, 305 (75.3%) experienced minimal depression, 68 (16.8%) had mild depression, and 32 (7.9%) had moderate depression in the past week (Table 2).

**Table 2:** Clinical characteristics of patients with DM 2 type at Adama Hospital Medical College, Oromia, Ethiopia, 2023 (*n* = 405).

Variable	Frequency	Percent (%)
Duration of follow-up in years		
<1	57	14.1
2–5	191	47.2
6–10	102	25.2
>11	55	13.6
BMI of participants		
Under weight	21	5.2
Normal weight	208	51.4
Over weight	153	37.8
Obese	23	5.7
Family history of DM		
No	405	64.2
Yes	145	35.8
Being a member of diabetic association		
No	327	80.7
Yes	78	19.3
Having Co morbid disease		
No	217	53.6
Yes	188	46.4
Treatment type		
Oral hypoglycemic	268	66.2
Insulin	17	4.2
Combination	120	29.6
Glycemia levels		
<126	130	32.1
≥126	275	67.9
Depression status		
Minimal depression	305	75.3
Mild depression	68	16.8
Moderate depression	32	7.9

### Food related factors and household food security status

Among the participants, 160 (39.5%) received nutrition education, while 338 (83.5%) reported difficulties accessing fruits and vegetables. Additionally, 331 (81.7%) were concerned about the high cost of food, and 159 (39.3%) had a poor understanding of the relationship between food and disease. A total of 272 (67.2%) and 200 (49.4%) are managed their diabetes through food planning and struggled with selecting appropriate foods, respectively. While, 336 (83%) of type 2 DM patients were living in households with food insecurity (Table 3).

**Table 3:** Food related factors and household food security status of patients with DM 2 type attending follow-up care at Adama Hospital Medical College, Oromia, Ethiopia, 2023 (*n* = 405).

Variable	Frequency	Percent (%)
Got nutrition education		
No	245	60.5
Yes	160	39.5
Difficult on choosing food		
No	205	50.6
Yes	200	49.4
Poor understanding on Food disease association		
No	246	60.7
Yes	159	39.3
Difficult on availability of fruits and vegetables		
No	67	16.5
Yes	338	83.5
Worrying about high cost of foods		
No	74	18.3
Yes	331	81.7
Control DM by food planning		
No	133	32.8
Yes	272	67.2
Ever attend DM education		
No	65	16.0
Yes	340	84.0
Food security status		
Food insecure	336	83
Food secure	69	17

### Psychosocial and behavioral conditions

**Figure 1:**
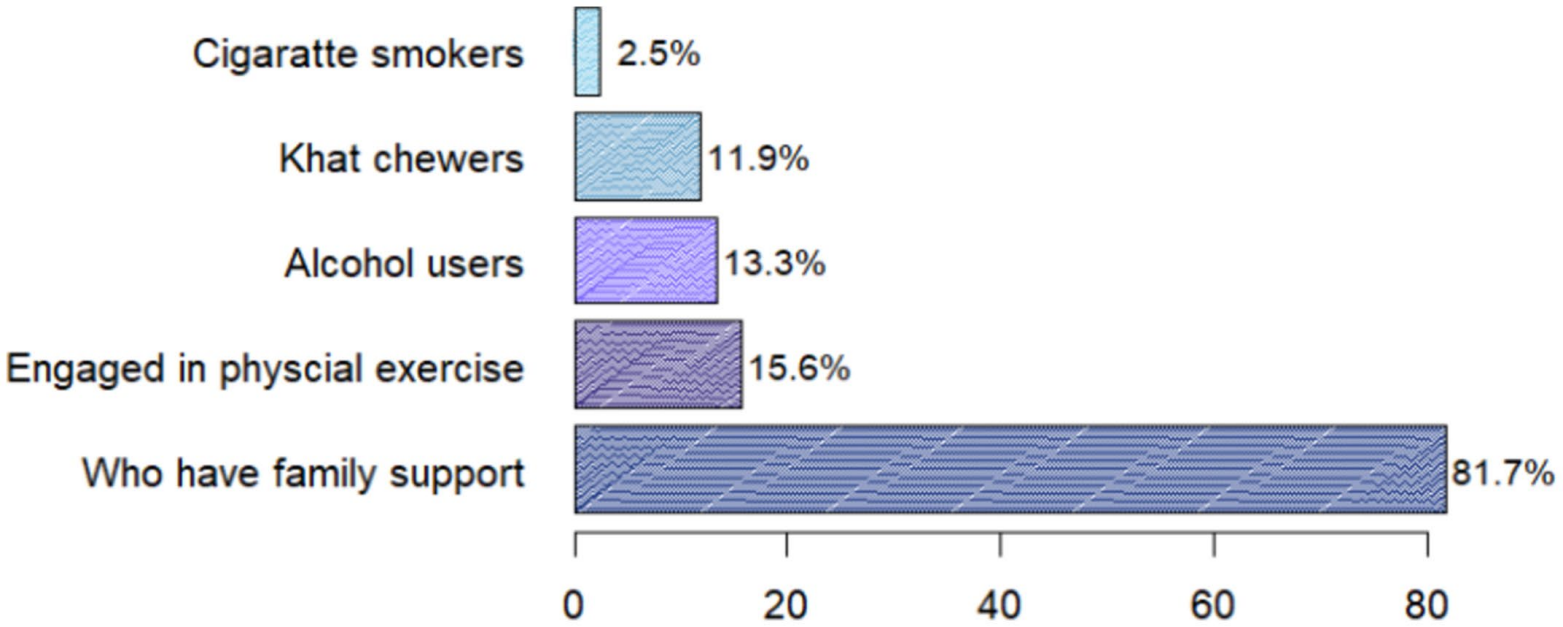
Psychosocial and behavioral conditions of type 2 diabetic patients attending follow-up care at Adama Hospital Medical College, 2023.

Of the total participants, 63 (15.6%) engaged in regular physical exercise, 54 (13.3%) use alcohol, 10 (2.5%) were smokers, and 74 (18.3%) participants experienced poor family support (Figure 1).

### Diabetic health belief

**Figure 2:**
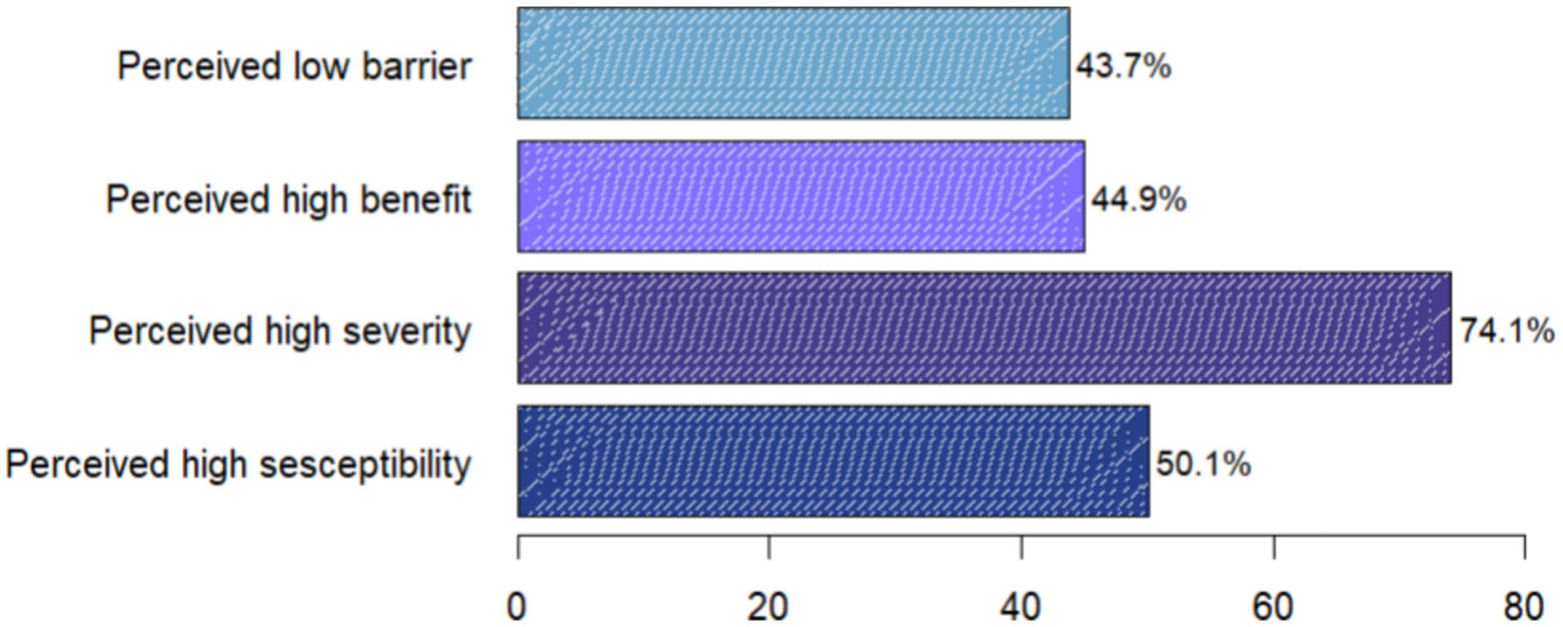
Diabetic health belief among type 2 diabetic patients attending follow up care at Adama Hospital Medical College, Ethiopia, 2023.

Regarding diabetic health belief, 203 (50.1%) of study subjects had a high perceived susceptibility, while 300 (74.1%) perceived the severity of their condition as high. One hundred seventy-seven (43.7%) of participants had low perceived barriers, while 182 (44.9%) recognized a high perceived benefit of the recommended diet (Figure 2).

### Magnitude of adherence to recommended diet

**Figure 3:**
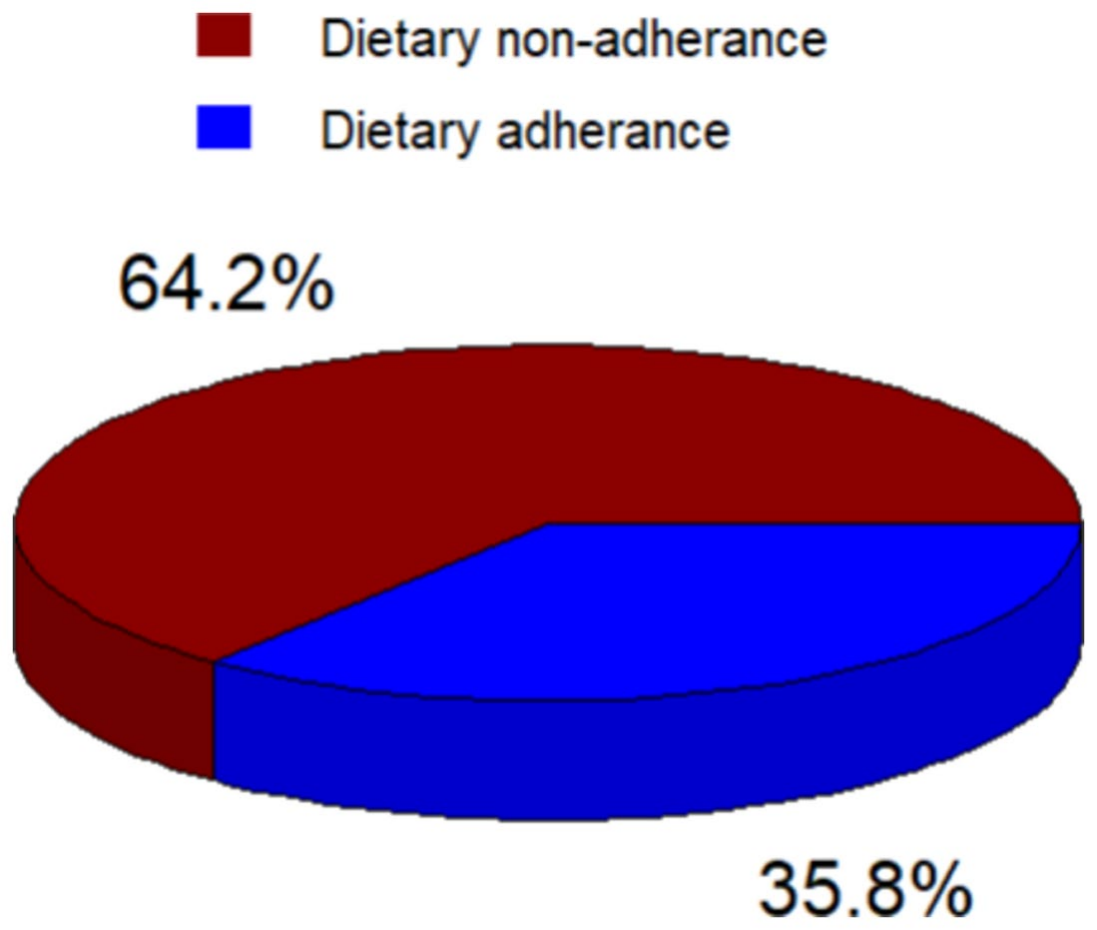
Dietary adherence status of type 2 diabetic patients attending follow up care at Adama Hospital Medical College, 2023.

The highest mean score was obtained for the question regarding consuming foods high in sugar and fat with a mean of 5.72 ± 1.14 and 6.3 4 ± 1.04 times a week, respectively. In this study, we found that the magnitude of non-adherence to the recommended diet among patients with type 2 diabetes mellitus was 64.2% (95% CI: 59.8, 68.6), based on the PDAQ (Figure 3).

### Factors associated with adherence to dietary recommendations of type 2 DM patients

In a multivariate logistic regression model, patients with low and middle incomes were 8.0 (AOR: 8.0; 95% CI: 3.4–19.2) times and 2.7 (AOR: 2.7; 95% CI: 1.47–5.13) times more likely to be non-adherent to the recommended diet, respectively, compared to patients with higher incomes. Patients with a glycemic level of 126 mg/dL or higher were 2.1 (AOR: 2.1; 95% CI: 1.17–3.94) times more likely to be non-adherent to the recommended diet. The odd of non-adherence to dietary recommendations was 12.7 (AOR: 12.7; 95% CI: 5.79–28.2) times higher among food-insecure patients compared to food-secure patients. Patients with low diabetic knowledge were 2.8 (AOR: 2.8; 95% CI: 1.49–5.55) times more likely to be non-adherent to the recommended diet. Regarding diabetic health belief, patients with low perceived susceptibility for diabetic were 2.9 (AOR: 2.9; 95% CI: 1.62–5.45) times more likely to be non-adherent to dietary recommendations compare to those with high perceived susceptibility (Table 4).

**Table 4:** Multivariable logistic regression analysis of the recommendation among dietary of patients with DM 2 type at Adama Hospital Medical College, Adama, Ethiopia, 2023 (*n* = 405).

Variables	Dietary adherence				
	Non-adherent	Adherent	COR (95% CI)	AOR (95% CI)	*P*-value
BMI category					
Underweight	92	16	1.09(0.29–4.40)	0.77(0.13–4.32)	0.701
Normal weight	89	44	1.05(0.41–2.69)	1.18(0.36–3.84)	0.432
Over weight	56	46	0.50(0.19–1.29)	0.62(0.19–2.03)	0.192
Obesity	23	39	1	1	
No of family size					
<5	171	106	1	1	
>5	89	39	1.42(0.90–2.21)	1.67(0.90–3.09)	0.231
Monthly income					
Low	98	14	11.04(5.63–21.55)	8.0(3.40–19.2)	**0.001**
Medium	117	60	3.07(1.89–5.00)	2.75(1.49–5.55)	**0.003**
High	45	71	1	1	
Treatment type					
Insulin	9	8	1	1	
Oral hypoglycemic	188	80	2.0(0.77–5.60)	1.61(0.41–6.24)	0.351
Combination	63	57	0.98(0.35–2.71)	1.09(0.27–4.33)	0.225
Member of diabetic association					
No	225	102	2.71(1.63–4.48)	1.57(0.76–3.25)	0.121
Yes	35	43	1	1	
Perceived susceptibility					
Low	169	33	6.30(3.96–10.0)	2.97(1.62–5.45)	**0.001**
High	91	112	1	1	
Perceived benefits					
Low	166	57	2.72(1.79–4.14)	1.64(0.92–2.99)	0.231
High	94	88	1	1	
Glycemia levels					
<126	54	76	1	1	
>126	206	69	4.20(2.69–6.54)	2.15(1.17–3.94)	**0.034**
Knowledge status					
Low	149	37	3.91(2.5–6.12)	2.88(1.49–5.55)	**0.026**
High	111	108	1	1	
Household food security status					
Food insecure	240	96	6.12(3.45–10.8)	12.7(5.79–28.2)	**0.001**
Food secure	20	49	1	1	
Depression status					
Minimal	185	120	1	1	
Mild	52	16	2.10(1.15–3.86)	2.05(0.92–4.54)	0.541
Moderate	23	9	1.65(0.74–3.70)	1.49(0.49–4.47)	0.132

NB, 1 = reference; COR, crude odds ratio; AOR, adjusted odds ratio; CI, confidence interval. Bold values are indicates statistically significant association with adherence to recommended diet.

## Discussion

This study assessed the extent of adherence to dietary recommendations among individuals with type 2 diabetes patients. The findings revealed that 64.2% of participants did not adhere to the recommended diet (95% CI: 59.8–68.6%). This finding is in line with similar studies conducted in Kuwait 63.5% (24), India 63% (25), and Mexico 62% (26), and in Jimma Medical Center, Ethiopia 64.3% (27). However, it is lower than the findings from studies conducted in Egypt (97.5%), Nepal (87.5%), Yemen (79%), and Debre Tabor General Hospital (74.3%) (9, 28–30). The findings of the current study are higher than those reported in studies conducted in Malaysia 40.8% (31) and Ethiopian teaching hospitals 55.7% (14). This discrepancy may be attributed to differences in socio-economic and cultural characteristics, study settings, study periods, sample sizes, and the types of foods available in different countries (14).

We found that 67.9% of the respondents had poor glycemic control, as indicated by elevated fasting blood glucose levels. This finding aligns with a study conducted in Arba Minch, Ethiopia (32). However, it is higher than results from studies conducted at Dilchora Referral Hospital in Dire Dawa, Eastern Ethiopia (54.7%) (12), another study in northwest Ethiopia (57.5%) (33), and a systematic review in Ethiopia (51%) (34). The discrepancy between our findings and those of other studies may be due to differences in the tests used to measure glycemic control-while the other studies utilized the recommended HbA1c test, our study relied on the fasting blood glucose (FBG) test. Additionally, variations in study participants and settings could also contribute to these differences.

The results of the current study revealed a strong association between low- and middle-income levels and non-adherence to dietary recommendations among patients with type 2 diabetes mellitus (T2DM). Patients with low income had 8.0 times higher odds of non-adherence, while those with middle income had 2.7 times higher odds compared to higher-income counterparts. This finding consistent with studies conducted in Nepal (29), Ethiopia (12, 22, 35). This likely due to financial constraints that limit access to a diverse range of foods necessary for meeting daily nutritional requirements (12). The financial limitation can result in reliance on cheaper, calorie-dense, and nutrient-poor foods, worsening dietary non-adherence and negatively affecting health outcomes.

In this study, non-adherence to the recommended diet was significantly higher among patients living in food-insecure households. The odds of non-adherence were 12.7 times greater for those in food-insecure households compared to those in food-secure households. This finding is consistent with studies conducted in the USA and Addis Ababa, Ethiopia (36, 37). Food insecurity negatively impacts overall dietary quality, making it difficult for food-insecure patients with diabetes to adhere to healthy diet recommendations. Evidence indicates that food-insecure diabetic patients consume fewer fruits, vegetables, and proteins, and face challenges in maintaining regular eating habits and following recommended diet plans (38).

We found that glycemic levels exceeding 126 mg/dL were significantly associated with adherence to the recommended diet among patients with type 2 diabetes mellitus. This finding is consistent with a study conducted in Ethiopia (12, 32, 33). This association may be justified as higher blood glucose levels often reflect inadequate dietary management and non-adherence to nutritional recommendations. When patients fail to follow a prescribed diet that includes the right portions of carbohydrates, fiber, and essential nutrients, their glycemic control can worsen. Adhering to a recommended diet is crucial for regulating blood sugar levels and promoting balanced nutrition.

This study found a significant association between low diabetes knowledge and non-adherence to dietary recommendations among T2DM patients. Participants with low knowledge about diabetes were 2.9 times more likely to not follow the recommended diet compared to those with better knowledge. This finding is consistent with studies conducted in Nepal, Egypt, and Dire Dawa, Ethiopia (22, 29, 39). This may be due to the fact that adequate knowledge about diabetes enables patients to understand the importance of dietary guidelines, thereby reducing confusion in managing their condition. As knowledge increases, adherence to dietary advice improves, while poor understanding leads to higher non-adherence (29).

Patients with low perceived susceptibility were 2.9 times more likely to not adhere to dietary recommendations compared to those with high perceived susceptibility. In this study, 50.1% of participants reported high perceived susceptibility, aligning with findings from a study in government hospitals in the Ilu Aba Bora zone, where 72.7% reported similar levels of perceived susceptibility (40). This may be because individuals who perceive themselves as less at risk are less motivated to follow dietary guidelines, underestimating the potential harm of poor eating habits and thus leading to higher non-adherence.

### Strength and limitation of the study

This study used the Perceived Dietary Adherence Questionnaire, a reliable and valid tool that is easy to administer and suitable for diverse populations, effectively assessing individuals’ perceptions of dietary adherence. Some limitations of the study include the following: First, the assessment of non-adherence to the recommended diet relied on self-reported data, which may be influenced by social desirability bias or recall issues, leading individuals to overestimate or underestimate their actual adherence. Second, the cross-sectional design limits the ability to establish temporal relationships between potential factors and dietary non-adherence. Finally, the absence of standardized practices for measuring carbohydrate spacing in the study context may limit the effectiveness of the assessment tool used.

## Conclusion

The magnitude of non-adherence to dietary recommendations among patients with diabetes mellitus type 2 (T2DM) was significantly higher than reported in previous studies. Key factors contributing to this non-adherence include low to middle income, high glycemic levels, food insecurity, limited diabetes knowledge, and low perceived susceptibility. To improve adherence among T2DM patients, it is essential to implement comprehensive diabetes education, address socioeconomic barriers by providing financial and food security support, and enhance patient awareness of dietary risks.

## Data Availability

The datasets presented in this study can be found in online repositories. The names of the repository/repositories and accession number(s) can be found in the article/supplementary material.
